# PD18 Cytomegalovirus Prophylaxis With Valganciclovir For HIV-Immunosuppressed Patients: Cost Effectiveness And Budget Impact Analysis From The Brazilian Healthcare System Perspective

**DOI:** 10.1017/S0266462324002848

**Published:** 2025-01-07

**Authors:** Ludmila Gargano, Ariane André, Isabela Freitas, Francisco de Assis Acurcio, Juliana Alvares-Teodoro, Augusto Guerra

## Abstract

**Introduction:**

Cytomegalovirus (CMV) infections are usually mild but can lead to serious consequences in immunocompromised patients, such as those infected with human immunodeficiency virus (HIV). Valganciclovir, which is approved for treating CMV, may be a cost-effective prophylactic for CMV disease in immunocompromised patients. Cost-effectiveness and budget impact analyses were conducted to compare valganciclovir prophylaxis with no prophylaxis in immunosuppressed patients with HIV.

**Methods:**

Immunosuppressed patients with HIV who were seropositive for CMV were modeled in a one-year horizon decision tree. Outcomes were occurrence of CMV end-organ disease (EOD)–retinitis and gastroenteritis (85 and 15 percent of cases, respectively)–and survival probability after EOD. Direct medical costs related to prophylaxis (induction and maintenance phases) and treatment of EOD were considered. The budget impact analysis (BIA) used the clinical and cost parameters of the cost-effectiveness analysis to compare scenarios with valganciclovir prophylaxis and no prophylaxis in the public health system over a five-year time horizon. The annual market share adoption rate used was 10 percent. The number of eligible patients was calculated from HIV and acquired immunodeficiency syndrome cases reported in the country and epidemiological estimates.

**Results:**

Valganciclovir prophylaxis had an incremental cost of BRL31,781 (USD6,531) and added 0.69 percent in one-year survival, resulting in an incremental cost-effectiveness ratio (ICER) of BRL4,610,022 (USD947,414) per death avoided in one year. The annual budget impact ranged from BRL35,660,931 (USD7,328,743) in the first year to BRL457,707,796 (USD94,064,365) in the fifth year, totaling BRL1,230,400,576 (USD252,861,870) over five years. In sensitivity analyses, the lack of a maximum treatment time for the maintenance phase with valganciclovir (50 to 365 days per year) resulted in a wide variation in the ICER and BIA results.

**Conclusions:**

Despite being recommended for the treatment of CMV retinitis, the use of valganciclovir in immunocompromised patients with HIV who are seropositive for CMV but have not developed CMV disease has a higher ICER when compared with no prophylaxis. For these patients, appropriate use of standard antiretroviral therapy to improve the immune system may be more appropriate.

